# End-stage kidney disease and rationing of kidney replacement therapy in the free state province, South Africa: a retrospective study

**DOI:** 10.1186/s12882-021-02387-x

**Published:** 2021-05-11

**Authors:** Thabang T Molaoa, Feziwe B Bisiwe, Kwazi CZ Ndlovu

**Affiliations:** 1 Division of Nephrology, Department of Internal Medicine, University of the Free State, Bloemfontein, South Africa; 2 Division of Nephrology and Hypertension, University of Cape Town, Cape Town, South Africa; 3 Kidney and Hypertension Research Unit, University of Cape Town, Cape Town, South Africa

**Keywords:** Incidence rate, End-stage kidney disease, Kidney replacement therapy, Dialysis access, South Africa

## Abstract

**Background:**

End-stage kidney disease (ESKD) and the required kidney replacement therapy (KRT) are significant public health challenges for low-and-middle-income countries. The South African government adopted a KRT rationing policy to balance the growing need for KRT and scarce resources. We aimed to describe the epidemiology and KRT access in patients with ESKD referred to the main public sector hospital in the Free State Province, South Africa.

**Methods:**

A retrospective study of adult patients with ESKD admitted to Universitas Academic Hospital for KRT, was conducted between 1 January 2016 and 31 December 2018. A review of the KRT committee decisions to offer or deny KRT based on the KRT rationing policy of the Free State was undertaken. Demographic information, KRT committee outcomes, laboratory test results, and clinical details were collected from assessment tools, KRT committee meeting diaries, and electronic hospital records.

**Results:**

Of 363 patients with ESKD referred for KRT access, 96 with incomplete records were excluded and 267 were included in the analysis. Median patient age was 40 (interquartile range, 33‒49) years, and male patients accounted for 56.2 % (150/267, *p* = 0.004) of the cohort. The average annual ESKD incidence was 49.9 (95 % confidence interval [CI], 35.8‒64.0) per-million-population. The most prevalent comorbidities were hypertension (42.3 %; 113/267), human immunodeficiency virus (HIV) (28.5 %; 76/267), and diabetes mellitus (19.1 %; 51/267). The KRT access rate was 30.7 % (82/267), with annual KRT incidence rates of 8.05 (95 % CI, 4.98‒11.1), 11.5 (95 % CI, 7.83‒15.1), and 14.1 (95 % CI, 10.3‒18.0) per-million-population in 2016, 2017, and 2018, respectively. Advanced organ dysfunction was the commonest reason recorded for KRT access denial (58.9 %; 109/185). Age (odds ratio [OR], 1.04; 95 % CI, 1.00‒1.07; *p* = 0.024) and diabetes (OR, 5.04; CI, 1.69‒15.03; *p* = 0.004) were independent predictors for exclusion from KRT, while hypertension (OR, 1.80; 1.06‒3.04; *p* = 0.029) independently predicted advanced organ dysfunction resulting in KRT exclusion.

**Conclusions:**

Non-communicable and communicable diseases, including hypertension, diabetes, and HIV, contributed to ESKD, highlighting the need for improved early prevention strategies to address a growing incidence rate. Two-thirds of ESKD patients were unable to access KRT, with age, diabetes mellitus, and advanced organ dysfunction being significant factors adversely affecting KRT access.

**Supplementary Information:**

The online version contains supplementary material available at 10.1186/s12882-021-02387-x.

## Background

Chronic kidney disease (CKD) defined by the persistent kidney damage or glomerular filtration rate (GFR) of < 60 mL/min/1.73 m^2^ is a significant contributor to mortality due to non-communicable diseases [1, 2]. Its prevalence increases with age, but in Sub-Saharan Africa (SSA), young adults tend to be disproportionately affected [3–5], with higher incidences of chronic glomerulonephritis, herbal/traditional remedies, and communicable diseases in SSA contributing to CKD’s overrepresentation among the younger groups [6, 7]. Accurate prevalence statistics in SSA are scarce, but an estimated prevalence rate of 13 % has been reported [8]. Low-to middle-income countries face many challenges in the prevention and early detection of CKD, leading to an increase in the risk of disease progression [8]. In the Republic of South Africa (RSA), CKD presents within a context of a rising double burden of communicable and non-communicable diseases [9, 10].

End-stage kidney disease (ESKD) is an advanced, irreversible final stage of CKD requiring kidney replacement therapy (KRT) to sustain life [1]. It is a public health challenge worldwide and poses an enormous economic burden on the healthcare systems [11, 12]. With a population of just more than a billion, SSA has 80 % of its population living on less than $2.5 a day [13]. Access to the full range of KRT options, including dialysis and kidney transplantation, is a challenge for most SSA countries due to the high costs, infrastructural constraints, and shortage of skilled personnel [14]. Estimates suggest a significant unmet need in Africa, projected to have the lowest KRT access rate in the world, ranging between 9 and 16 % [14]. Furthermore, most dialysis centres are located in urban areas creating internal disparities in access to a full range of ESKD management options [15]. This situation favours access to KRT by those in urban areas with the ability to fund treatment costs.

In RSA, those who can afford private insurance, approximately 16 % of the population, receive a full range of KRT modalities. At the same time, the majority who are mostly indigent rely on the public sector [16]. Thus, the RSA government adopted a KRT rationing policy for the public sector based on criteria that select patients considered to be the best candidates for transplantation to balance the growing need for KRT and the scarce resources available [17]. Various regions of the country have adapted local variants of this rationing policy, reflecting local resource constraints. These rationing policies’ ability to enable KRT access based on transplantability and potential to reinforce existing socioeconomic inequalities have been controversial. Furthermore, there are scarce data on the ESKD incidence in many SSA countries and the extent to which the KRT need is satisfied. Our study aimed to define the profile of the patients needing KRT, the KRT access rate, and the factors associated with the denial of KRT access in the main public sector hospital of the Free State (FS) Province, RSA.

## Methods

### Patients and study design

In this retrospective study, we reviewed patients’ records that were referred for KRT and were evaluated by the KRT committee at Universitas Academic Hospital (UAH) from 1 January 2016 to 31 December 2018. Patients evaluated by the KRT committee included those with CKD stages 4 and 5 according to eGFR and those assessed by the referring health care practitioner to need dialysis due to symptoms or biochemical abnormalities attributed to chronic renal failure. UAH is an academic hospital located in Bloemfontein, RSA, and provides tertiary services to the FS province. The kidney unit at UAH receives referrals for KRT from public sector health care facilities throughout the FS province. The FS province is a predominantly rural province with 2.9 million people according to the 2018 estimates, and it covers an area of 129 825 km², with approximately 85 % of the population served by the public health sector [18, 19]. The province has 17 dialysis centres: 11 privately owned centres serving those with health insurance and 6 public sector dialysis centres coordinated centrally by the UAH. The FS public health care sector served an estimated 2.36, 2.44, and 2.48 million people without medical insurance in 2016, 2017, and 2018, respectively [19, 20]. The FS KRT programme offers peritoneal dialysis (PD) preferentially to patients starting dialysis and haemodialysis to patients unable to undergo PD. At the same time, kidney transplantation is limited to patients with live related donors who can travel to neighbouring provinces for transplantation.

**Fig. 1:**
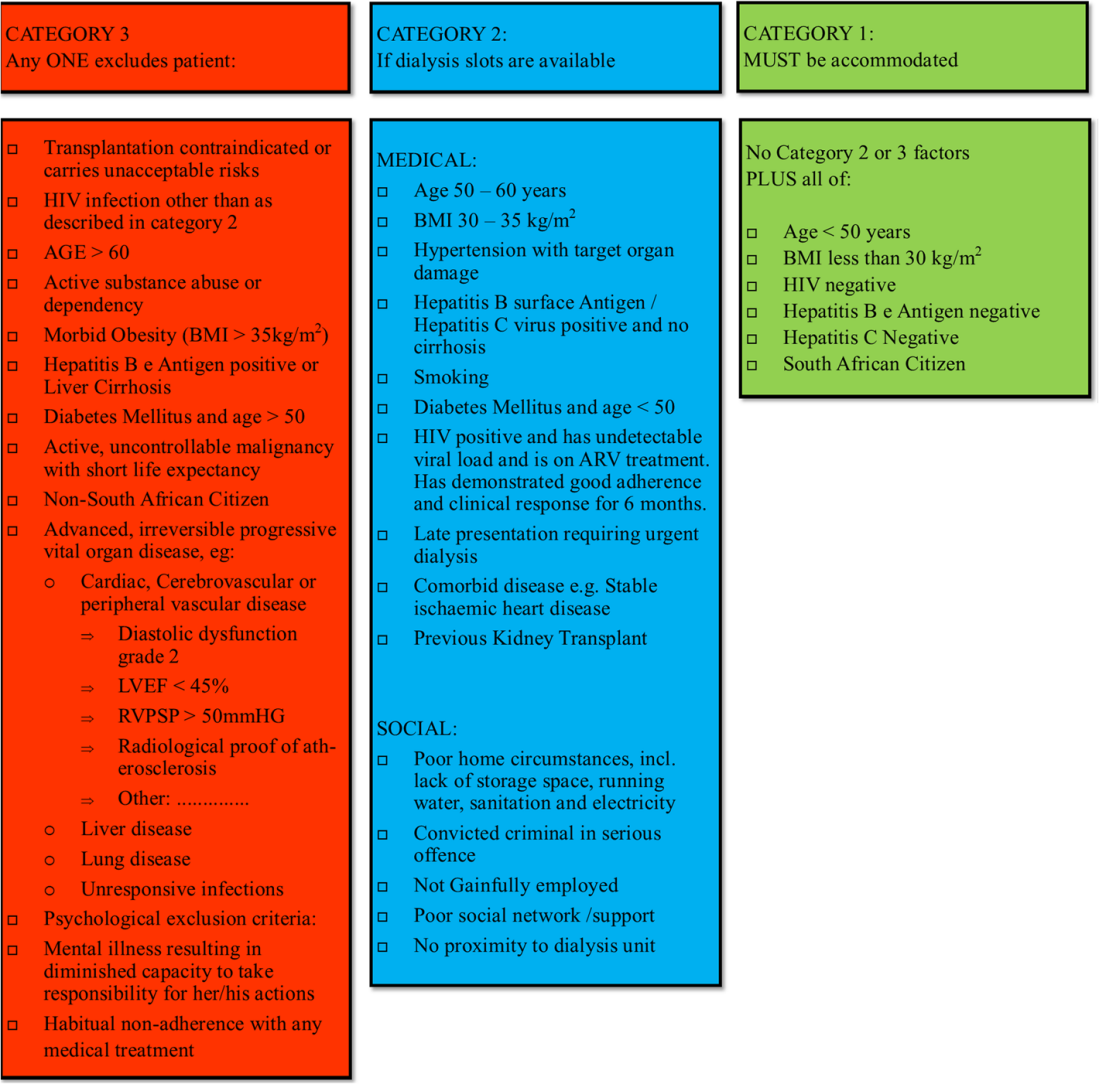
Free State chronic kidney replacement therapy guidelines. ARV, antiretroviral; BMI, body mass index; HIV, human immunodeficiency virus; LVEF, left ventricular ejection fraction; RVPSP, right ventricular peak systolic pressure

The UAH KRT committee, which comprises hospital managers, nephrologists, attending medical officers, social workers, dieticians, and nursing staff, meet weekly to discuss dialysis-requiring patients referred to the unit. Patients are assessed for KRT eligibility according to the FS KRT guidelines [21]. Patient considered as not the best candidates for kidney transplantation are classified as category 3 and are not offered KRT. Those with comorbidities that are manageable and potentially reversible are classified as category 2 and are offered KRT when dialysis resources are available. Category 1 includes young patients considered good transplant candidates with minimal comorbidities and are prioritised for KRT (Fig. 1).

The diaries containing minutes of the KRT committee meetings, the assessment forms, national health laboratory service electronic results, and electronic hospital records (MEDITECH) were reviewed to obtain the names and demographic details, KRT committee discussion outcomes, laboratory test results, and clinical information. The study population comprised adult patients (≥ 18 years old) diagnosed with ESKD and evaluated by the UAH KRT committee between 2016 and 2018. Patients with incomplete data in the assessment tool documents and clinical and socio-demographic records were excluded.

All category 3 patients were denied KRT access according to the FS KRT policy, and both categories 1 and 2 were deemed acceptable for KRT access. Criteria for exclusion from KRT access included concomitant irreversible advanced organ dysfunction. Cardiac disease was assessed using an echocardiogram and defined as an ejection fraction of < 45 % or diastolic dysfunction grade 2 or higher. Significant vascular disease was defined by any vascular calcifications that can be visualised on X-ray. Pulmonary hypertension was defined as a right ventricular peak systolic pressure of > 50 mmHg. Other classifying criteria are depicted in Fig. 1.

The incidence rates were calculated as the number of new cases divided by the provincial population served by the public health care sector as published by Statistics South Africa for that period. The rates were expressed as per million population (pmp).

### Statistical analysis

Continuous variables are summarised using medians and interquartile ranges and compared using the Wilcoxon-Mann-Whitney test. Kruskal-Wallis equality-of-populations rank test was used for comparing more than two continuous variables. Categorical and ordinal variables are summarised using proportions and percentages and compared using Pearson’s chi-square test or Fisher’s exact test, as appropriate. Logit transform was used to calculate confidence intervals of proportions and differences compared using a two-sample test for proportions. Univariate logistic regression was used to estimate the association between exclusion from the KRT programme and various risk factors. Multivariable logistic regression analysis was used to identify independent predictors for exclusion from the KRT programme. Covariates included in the logistic regression model were age, sex, employment status, presence of dependants, poor home circumstances as determined by a social worker, diabetes, hypertension, human immunodeficiency virus (HIV) status, history of smoking, and serum creatinine level. All analyses were performed using Stata Statistical Software, Release 15 (StataCorp, College Station, TX, US), and the level of significance was set at p < 0.05.

## Resutls

### Patient Characteristics and Demographics

From January 2016 to December 2018, a total of 363 patients were assessed for KRT eligibility at the UAH, 96 patient files were excluded for incomplete information, leaving 267 files for further analysis. Only one third (30.7 %; 82/267) of the patients with ESKD, referred for KRT, were offered dialysis, while the rest (69.3 %; 185/267) were denied KRT access. The median age of the patients in our study was 40 (interquartile range [IQR], 33‒49) years. The median age among the patients that were denied KRT access was 43 (IQR 34‒51) compared to 37 (IQR 30‒43) years among those accepted for KRT (*p* < 0.001). There were significantly more male patients (56.2 %; 150/267) than female patients (43.8 %; 117/267) (*p* = 0.004) in the study cohort. The proportion of employed patients was 47.6 % (126/267), while 44.9 % (119/267) were unemployed, and 6.8 % (18/267) were pensioners. Those with dependants made up 75.5 % (200/267) of our study population, while 21.9 % had no dependants (*p* < 0.001). Only 5.6 % (15/267) of the patients admitted to smoking and alcohol use, which was significantly higher among those excluded from KRT access (7.6 % vs. 1.2 %; *p* = 0.043). According to the social worker assessment, slightly less than 2 % of the patients were considered to have poor home circumstances and poor social support networks (Table 1).

**Table 1:** Background characteristics of end-stage kidney disease patients presenting for kidney replacement therapy

	KRT Accepted (*n* = 82)	KRT excluded (*n* = 185)	*p*-value	Total (%, 95 % CI)	*p*-value
Age (years), median (IQR)	37 (30–43)	43 (34–51)	< 0.001	40 (33–49)	
Sex, n (%)					
Male	46 (56.1)	104 (56.2)	0.986	150 (56.2; 50.0–62.0)	0.004
Female	36 (43.9)	81 (43.8)		117 (43.8; 38.0–49.9)	
Employment, n (%)					
Employed/Self employed	40 (49.4)	86 (46.7)	0.691	126 (47.6; 41.6–53.6)	Reference
Unemployed	40 (49.4)	79 (42.9)	0.331	119 (44.9; 39.0–51.0)	0.542
Pensioner	0	18 (9.8)	0.002	18 (6.8; 4.3–10.5)	< 0.001
Unspecified	1 (1.2)	1 (0.5)	0.519	2 (0.8; 0.1–3.0)	< 0.001
Marital status, n (%)					
Married	41 (50.6)	105 (57.1)	0.331	146 (55.1; 49.0–61.0)	Reference
Single	40 (49.4)	71 (38.6)	0.101	111 (41.9; 36.1–47.9)	0.002
Divorced	0	6 (3.3)	0.182	6 (2.3; 1.0–5.0)	< 0.001
Unknown	0	2 (1.1)	1.000	2 (0.8; 0.1–3.0)	< 0.001
Dependants, n (%)					
Yes	56 (69.1)	144 (78.3)	0.112	200 (75.5; 70.0–80.3)	Reference
No	21 (25.9)	37 (20.1)	0.291	58 (21.9; 17.3–27.3)	< 0.001
Unknown	4 (4.9)	3 (1.6)	0.206	7 (2.6; 1.3–5.4)	< 0.001
Citizenship, n (%)					
South African	80 (97.6)	178 (96.2)	0.726	258 (96.6; 93.6–98.2)	< 0.001
Non-South African	2 (2.4)	7 (3.8)		9 (3.4; 1.8–6.4)	
Smoking and/or alcohol, n (%)	1 (1.2)	14 (7.6)	0.043	15 (5.6; 3.4–9.1)	
Illicit substance abuse, n (%)	0	2 (1.1)	1.000	2 (0.8; 0.2–3.0)	
Social factors, n (%)					
Poor home circumstances	1 (1.2)	4 (2.2)	0.548	5 (1.9; 0.8–4.4)	1.000
Poor social network/support	0	5 (2.8)		5 (1.9;0.8–4.4)	

*CI* confidence interval; *IQR* interquartile range; *KRT* kidney replacement therapy

*Illicit substance abuse includes regular use of cannabis and other illegal substances

### Clinical characteristics

Among the patients with ESKD included in the study, the most prevalent comorbidities were hypertension (42.3 %; 113/267), HIV (28.5 %; 76/267) and diabetes mellitus (19.1 %; 51/267). Likewise, the three most significant causes of ESKD were hypertension (32.2 %; 86/267), HIV-associated nephropathy (15.7 %; 42/267) and diabetes mellitus (12.4 %; 33/267). Glomerulonephritis was documented as the cause of ESKD in 9 % of the study patients. The median serum creatinine at presentation was 890 (IQR, 670‒1210) µmol/L, with the accepted group having a higher median (1044.5; IQR 807‒1403 µmol/L) than that in the denied group (794; IQR, 587‒1143 µmol/L), *p* < 0.001 (Table 2).

**Table 2:** Clinical characteristics

	KRT Accepted (*n* = 82)	KRT excluded (*n* = 185)	*p*-value	Total (%, 95 % CI)	*p*-value
Hypertension, n (%)	36 (43.9)	77 (41.6)	0.728	113 (42.3, 36.5–48.4)	Reference
Diabetes, n (%)	4 (4.9)	47 (25.4)	< 0.001	51 (19.1, 14.8–24.3)	< 0.001
SLE, n (%)	6 (7.3)	6 (3.2)	0.138	12 (4.5, 2.6–7.8)	< 0.001
Hepatitis B, n (%)	1 (1.2)	4 (2.2)	1.000	5 (1.9, 0.7–4.4)	< 0.001
Hepatitis C, n (%)	1 (1.2)	1 (0.5)	0.521	2 (0.8, 0.2–3.0)	< 0.001
HIV status, n (%)					
Negative	61 (74.4)	113 (61.1)	Reference	174 (65.2, 59.2–70.7)	
Positive	21 (25.6)	55 (29.7)	0.491	76 (28.5, 23.4–34.2)	0.001
Unknown	0	17 (9.2)	0.002	17 (6.4, 4.0–10.0)	
Kidney disease aetiology, n (%)					
Hypertension	27 (32.9)	59 (31.9)	0.867	86 (32.2, 26.8–38.1)	Reference
Diabetes mellitus	1 (1.2)	32 (17.3)	< 0.001	33 (12.4, 8.9–16.9)	< 0.001
Glomerulonephritis	11 (13.4)	13 (7.0)	0.092	24 (9.06.1–13.1)	< 0.001
Lupus nephritis	6 (7.3)	6 (3.2)	0.138	12 (4.5,2.6–7.8)	< 0.001
HIVAN	14 (17.1)	28 (15.1)	0.688	42 (15.7, 11.8–20.6)	< 0.001
FSGS	1 (1.2)	2 (1.1)	1.000	3 (1.1, 0.3–3.4)	< 0.001
Autosomal Polycystic kidney disease	6 (7.3)	5 (2.7)	0.098	11 (4.1, 2.3–7.3)	< 0.001
Connective tissue diseases/Vasculitis	0	5 (2.7)	0.328	5 (1.9, 0.8–4.4)	< 0.001
Obstructive uropathy	1 (1.2)	10 (5.4)	0.181	11 (4.1, 2.3–7.3)	< 0.001
Other^a^	2 (2.4)	7 (3.8)	0.726	9 (3.4, 1.8–6.4)	< 0.001
Unknown	13 (15.9)	18 (9.7)	0.150	31 (11.6, 8.3–16.1)	< 0.001
Serum creatinine (µmol/L), median (IQR)	1044.5 (807–1403)	794.5 (587–1143)	< 0.001	890 (670–1210)	

*CI* confidence interval; *ESKD* end-stage kidney disease; *FSGS* focal segmental glomerulosclerosis; *HIV* human immunodeficiency virus; *HIVAN* HIV associated nephropathy; *IQR* interquartile range; pmp, per-million-population; *KRT* kidney replacement therapy; *SLE* systemic lupus erythematosus

^a^Other causes included oxalosis, multiple myeloma, reflux nephropathy, hypoplastic kidneys and renal agenesis

### ESKD incidence and KRT rates

The ESKD incidence rate was 26.7 pmp in 2016, 41.4 pmp in 2017, and 41.6 pmp in 2018, with a cumulative incidence rate of 110.1 pmp over these three years. KRT access rates were 30.2 % (19/63), 27.7 % (28/101), and 34.0 % (35/103) of study patients referred for KRT access in 2016, 2017 and 2018, respectively (*p* = 0.622). When all 363 patients referred for KRT at the UAH between 2016 and 2018 were considered, the cumulative ESKD incidence rate was 149.7 (95 % CI, 125.2‒174.1) pmp with an estimated average annual ESKD incidence of 49.9 (95 % CI, 35.8‒64.0) pmp. A steady increasing trend of KRT incidence rate was observed, starting at 8.05 (95 % CI, 4.98‒11.1) in 2016, 11.5 (95 % CI, 7.83‒15.1) in 2017 and 14.1 (95 % CI, 10.3‒18.0) in 2018 (Table 3). The KRT incidence increased by 75 % between 2016 and 2018 (*p* = 0.045).

**Table 3:** Access to kidney replacement therapy

	2016 (*n* = 63)	2017 (*n* = 101)	2018 (*n* = 103)	*p*-value	Total (%, 95 % CI) (*n* = 267)	*p*-value
ESKD incidence rate, pmp (95 % CI)	26.7 (20.0–33.4)	41.4 (33.2–49.6)	41.6 (33.4–49.8)	0.006^a^ 0.005^b^	110.1 (96.6–123.6)	
Accepted for dialysis, n (%)	19/63 (30.2 %; 20.0–42.8 %)	28/101 (27.7 %; 19.8–37.3 %))	35/103 (34.0 %; 25.4–43.7 %))	0.622	82/267 (30.7 %; 25.4–36.5 %))	
KRT incidence, pmp (95 % CI)	8.05 (4.98–11.1)	11.5 (7.83–15.1)	14.1 (10.3–18.0)	0.230^a^ 0.045^b^	33.8 (27.7–39.9)	
Age (years), median (IQR)	36 (32–48)	43 (35–50)	40 (31–48)	0.131	40 (33–49)	
Hypertension, n (%)	27/63 (42.8)	50/101 (49.5)	36/103 (35.0)	0.109	113/267 (42.3, 36.5–48.4)	
Diabetes, n (%)	13/63 (20.6)	21/101 (20.8)	17/103 (16.5)	0.693	51/267 (19.1, 14.8–24.3)	
HIV, n (%)	13/63 (20.6)	30/101 (29.7)	33/103 (32.0)	0.270	76/267 (28.5, 23.4–34.2)	
Serum creatinine (µmol/L), median (IQR)	807 (529–1050)	995.5 (708–1050)	883 (650–1184)	0.033	890 (670–1210)	
**Reason for exclusion from KRT programme**						
Concomitant organ dysfunction, n (%)	25/44 (56.8)	45/73 (61.6)	39/68 (57.4)	0.830	109/185 (58.9; 51.6–65.8)	Reference
Cardiac disease, n (%)	9/44 (20.4)	16/73 (21.9)	22/68 (32.4)	0.250	47/185 (25.4;19.6–32.2)	Reference
Vascular disease, n (%)	7/44 (20.4)	8/73 (23.3)	12/68 (16.2)	0.511	27/185 (14.6; 10.2–20.5)	0.009
Pulmonary hypertension, n (%)	9/44 (20.4)	17/73 (23.3)	11/68 (16.2)	0.571	37/185 (20.0; 14.8–26.4)	0.215
Lung disease, n (%)	1/44 (2.3)	5/73 (6.8)	2/68 (2.9)	0.467	8/185 (4.3; 2.2–8.4)	< 0.001
Noncompliance, n (%)	4/44 (9.1)	2/73 (2.7)	4/68 (5.9)	0.286	10/185 (5.4; 2.9–9.8)	< 0.001
Psychosocial, n (%)	2/44 (4.6)	2/73 (2.7)	4/68 (5.9)	0.660	8/185 (4.3; 2.2–8.4)	< 0.001
Substance abuse, n (%)	2/44 (4.6)	4/73 (5.5)	1/68 (1.5)	0.483	7/185 (3.8; 1.8–7.8)	< 0.001
Diabetes and age ≥ 50 years, n (%)	3/44 (6.8)	9/73 (12.3)	2/68 (2.9)	0.124	14/185 (7.6; 4.5–12.4)	< 0.001
Uncontrolled HIV^c^, n (%)	3/44 (6.8)	9/73 (12.3)	9/68 (13.2)	0.610	21/185 (11.4; 7.5–16.8)	< 0.001
Age ≥ 60 years, n (%)	4/44 (9.1)	7/73 (9.6)	6/68 (8.8)	1.000	17/185 (9.2; 5.8–14.3)	< 0.001
BMI ≥ 35 kg/m^2^, n (%)	3/44 (6.8)	3/73 (4.1)	7/68 (10.3)	0.357	13/185 (7.0; 4.1–11.8)	< 0.001
Other, n (%)	2/44 (4.6)	3/73 (4.1)	4/68 (5.9)	0.910	9/185 (4.9; 2.5–9.1)	< 0.001

*BMI* body mass index; *CI* confidence interval; *ESKD* end-stage kidney disease; *HIV* human immunodeficiency virus; *IQR* interquartile range; *pmp* per-million-population; *KRT* kidney replacement therapy

^a^Comparison of 2016 and 2017 incidence rates

^b^Comparison of 2016 and 2018 incidence rates

^c^Uncontrolled HIV refers to unsuppressed viral load

### Reasons for non-admission to the KRT program

The main reasons for being denied KRT access in the FS public sector are listed in Table 3. Medical factors accounted for the largest proportion of reasons to be denied KRT, including communicable and non-communicable diseases. Concomitant advanced organ dysfunction was the most typical medical cause of denial (58.9 %; 109/185), including cardiac disease (25.4 %; 47/185), vascular disease (14.6 %; 27/185), chronic lung disease (4.3 %; 8/185), among others. Uncontrolled HIV infection (11.4 %; 21/185) and age above 60 years (9.2 %; 17/185), respectively, were other prominent exclusion factors. Behavioural and psychosocial factors, such as habitual non-compliance to medical treatment, substance abuse, and mental illness, were cited as contributing to 13.5 % of the exclusions combined. Diabetes mellitus was a significantly higher comorbidity among those denied KRT compared to that in patients who were offered KRT (25.4 vs. 4.9 %; *p* < 0.001).

Age (Odds Ratio [OR], 1.04; 95 % CI, 1.00‒1.07; *p* = 0.024) and diabetes (OR, 5.04; CI, 1.69‒15.03; *p* = 0.004) were independent predictors for non-acceptance to the KRT programme. However, hypertension (OR, 1.80; 1.06‒3.04; *p* = 0.029) independently predicted concomitant advanced organ dysfunction resulting in exclusion from the KRT programme (Table 4).

**Table 4:** Univariate and multivariable logistic regression for exclusion from KRT programme

	Univariate logistic regression		Multivariable logistic regression	
	**Odds Ratio (95 % CI)**	***p*-value**	**Odds Ratio (95 % CI)**	***p*-value**
**All-cause exclusion from KRT programme**				
Age	1.04 (1.02–1.07)	< 0.001	1.04 (1.00–1.07)	0.024
Sex	1.00 (0.59–1.68)	0.986	0.86 (0.47–1.56)	0.618
Employment status	1.31 (0.86–1.99)	0.202	1.57 (0.96–2.59)	0.075
Has dependents	1.61 (0.89–2.89)	0.113	0.94 (0.41–2.17)	0.888
Poor home circumstances	3.35 (0.59–19.02)	0.172	3.07 (0.50–18.69)	0.224
Diabetes	6.64 (2.31–19.13)	< 0.001	5.04 (1.69–15.03)	0.004
Hypertension	0.98 (0.56–1.70)	0.937	0.97 (0.52–1.78)	0.910
HIV	1.23 (0.68–2.21)	0.492	1.12 (0.57–2.20)	0.738
Smoking and alcohol	6.63 (0.86–51.3)	0.070	4.56 (0.56–36.97)	0.155
Serum creatinine	1.00 (1.00–1.00)	0.001	1.00 (1.00–1.00)	0.007
**Exclusion due to advanced organ dysfunction.**				
Age	1.00 (0.98–1.02)	0.673	1.00 (0.98–1.02)	0.811
Sex	0.79 (0.48–1.29)	0.345	0.83 (0.49–1.40)	0.486
Hypertension	1.76 (1.07–2.88)	0.026	1.80 (1.06–3.04)	0.029
Diabetes	1.67 (0.90–3.08)	0.103	1.65 (0.85–3.20)	0.136
HIV	0.58 (0.33–1.01)	0.054	0.58 (0.32–1.05)	0.070
Smoking and alcohol	2.28 (0.79–6.60)	0.129	1.97 (0.65–5.95)	0.228
Serum creatinine	1.00 (1.00–1.00)	0.005	1.00 (1.00–1.00)	0.005

*CI* confidence interval; *HIV* human immunodeficiency virus; *KRT* kidney replacement therapy

## Discussion

This study describes the epidemiology and KRT access of patients with ESKD referred for dialysis in the public health sector of the FS province, a predominantly rural province in South Africa. An average annual ESKD incidence of 49.9 pmp was estimated, with 30 % of those presenting with ESKD able to access KRT. Hypertension, diabetes, and HIV were the commonest comorbidities, while advanced organ dysfunction and uncontrolled HIV were the leading reasons for denying KRT access. Age and diabetes mellitus were independent predictors for denial of KRT access.

The crude annual incidence rates of ESKD increased from 26.7 to 41.6 pmp between 2016 and 2018. However, the crude average annual ESKD incidence rate was 49.9 pmp when the entire cohort of 363 patients was considered, including the 96 patient records excluded from analysis due to incomplete data. Still, this is likely underestimating the true ESKD incidence rate in the FS since some patients were not referred to the UAH, particularly if they had an obvious exclusion factor. The ESKD incidence reported in the literature is highly variable, ranging from 89 pmp in some western countries such as Finland to 363 pmp in the United States of America [22]. In Latin America, a high incidence rate of 458 pmp has been reported in Mexico [23]. There is a dearth of reported ESKD incidence rates in Africa due to the limited access to KRT and poor data collection mechanisms [13]. With relatively non-restrictive KRT access and a high prevalence of associated non-communicable diseases, Libya reported an ESKD incidence of 282 pmp [24]. These higher incidence rates than those estimated in this study suggest a significant underestimation of ESKD incidence in RSA, as a substantial number of patients with ESKD are palliated at the primary health care level without referral to a specialised kidney unit.

A typical patient with ESKD referred for KRT was young (mean age of 40 years old), married with dependents, and of male preponderance. This young profile was primarily attributed to the KRT policy excluding those with age above 60 years and age above 50 years in people with diabetes, thus influencing the likelihood of referral from primary health care levels. Other contributors included the favourable consideration of society’s economically active members. Furthermore, age was a significant predictor of KRT access, with those offered KRT access being significantly younger than those excluded (37 vs. 43 mean years old, *p* < 0.001). Moreover, the increased likelihood of having comorbidities and advanced organ damage with progressing age was another reason for the age-associated bias. Typically, advancing age is associated with an increased incidence of ESKD [22, 25]. Thus, the age criterion likely contributed to a significant undermining of the ESKD incidence, since elderly patients are being censored at the primary health care level.

Regarding behavioural factors, the use of alcohol and smoking was significantly higher in the excluded group. According to the program guidelines, no one was excluded from the KRT program solely based on smoking or alcohol use. Nonetheless, these behavioural factors were present disproportionally in some patients excluded due to advanced organ dysfunction and substance abuse, including alcohol abuse. Social factors such as poor home circumstances and social support networks were not significantly higher in the excluded group. However, Cape Town reports reviewing the KRT programme in the Western Cape (WC) province, RSA, showed a significant influence of psychosocial factors in denying KRT access [11, 26]. This incongruity highlights differing tolerances and thresholds in evaluating such highly subjective social variables among the country’s different regions. The FS is predominantly a rural province with a significant indigent population and a gross domestic product lower than half that of WC. Nearly half of the adults in FS live below $2.5/day compared to a third in the WC [27]. Thus, it is conceivable that the FS employed a much more accommodative approach to determine what constitutes poor home circumstances and social support compared to that in the WC. A more liberal outlook would accommodate the expected higher proportion of FS residents living under rural and poor socioeconomic conditions.

At the time of the KRT eligibility assessment, the mean creatinine level was documented as 890 µmol/L, with the accepted group having a significantly higher creatinine level than that in the excluded group (1044 vs. 794 µmol/L, p < 001). The lower creatinine levels among the excluded group were likely an artefact of non-referral of those with severe kidney failure and an obvious exclusion factor. These would have been palliated at the primary health care level if there were no suggestions of acute kidney failure. Those with suspicion of acute kidney disease were offered dialysis for up to 6 weeks. High levels of creatinine at referral suggest a prevailing problem of late presentation of patients with advanced CKD to the nephrology unit. Failure of early identification of CKD and timely referral for tertiary kidney care highlights a primary health care deficiency in the FS public health care system and RSA. A late referral is associated with adverse morbidity and mortality outcomes leading to unfavourable socioeconomic consequences, which can be counterproductive in a resource-limited environment [28, 29]. A shortage of nephrologists, most of whom are concentrated in urban centres, and poor awareness of CKD among primary health care providers are likely underlying factors that contribute to the late referral of ESKD [15, 30]. The diverse and immense patient burdens faced by the few primary health care providers in rural and peri-urban health care centres constitute another possible contributing factor to inadequate surveillance and timely identification of CKD.

Prevention and early CKD identification are particularly important among high-risk groups such as those with hypertension, diabetes, and HIV [31]. Screening for proteinuria and reductions in GFR are cost-effective measures that allow for early diagnosis and intervention to reduce the risk of ESKD and the associated cardiovascular events and mortality [32]. Although population-wide urinalysis screening of working adults and school-aged children aided in lowering the incidence of glomerulonephritis and ESKD in Japan [33], the cost-effectiveness of mass screening strategies in resource-limited low-to-middle-income countries is controversial [31, 34]. Both primary and secondary prevention strategies depend on the available local resources [35]. Adequately trained primary healthcare personnel who can recognise and manage both communicable and non-communicable diseases is critical for a sustainable prevention strategy. The RSA public health sector is fortunate to have free and general availability of major drugs needed to manage hypertension, diabetes, HIV, and other common disorders. This essential drug availability includes drugs vital to early CKD care, such as inhibitors of the renin-angiotensin-aldosterone system. However, shortages of appropriately capacitated human capital are crucial barriers that impede the success of prevention strategies in the FS and RSA as a whole [15]. Investing in the capacitation of the public health care system and promoting clinical practice standards that encourage regular screening and initiation of early interventions against kidney injury, communicable diseases, and non-communicable diseases, among others, are vital in mitigating the risk posed by ESKD.

Both communicable and non-communicable diseases were associated with the reported cause of kidney disease in our cohort. Hypertension and HIV were the most prevalent comorbidities and commonest attributed causes of ESKD, with no significant differences between the accepted and the non-accepted groups. Likewise, hypertension has been reported as the commonest cause of ESKD among those receiving KRT in the RSA [36]. Furthermore, hypertension has been noted to be the foremost cause of kidney disease in SSA [13]. However, physician-reported ESKD causes attributed to hypertension are notoriously unreliable, as hypertension is both a cause and a consequence of ESKD, especially when the presentation is late and without the benefit of a kidney biopsy. By contrast, diabetes has been reported to be the most common cause of ESKD in both United States (USRDS) and European (ERA-EDTA) kidney registries [25, 37]. In our cohort, diabetes mellitus was the third-commonest cause of ESKD and was significantly more common among the excluded group than among the KRT-offered group. However, this result was likely influenced by the non-referral sorting of patients with diabetes who are older than 50 years, as it is a recognised exclusion parameter. This bias likely resulted in an underappreciation of diabetes-associated kidney disease in our cohort. The prevalence of diabetes and its associated complications such as kidney diseases is expected to increase, with SSA projected to have the largest increase [38, 39]. As diabetes and the related ESKD are linked to significant cardiovascular morbidity and mortality, considerable associated socioeconomic and public health burdens can be imposed on societies who can least afford the additional burden [40, 41].

Given the shortage of dialysis machines and health care personnel and the high cost associated with KRT, the FS KRT rationing programme attempts to accommodate those who can potentially benefit the most from KRT. During the study, the KRT incidence increased by 75 %, from 8.05 pmp in 2016 to 14.2 pmp in 2018. However, the acceptance rate did not change significantly during the same period, with two-thirds of the referred patients with ESKD not being offered dialysis. Even with the reported underestimated burden of ESKD, it is evident that the FS KRT program cannot support the indigent population’s KRT needs. A systemic review by Liyanage et al. estimated Africa to have the lowest KRT access rates in the world, ranging from 9 to 16 % [14]. Considering those who were not referred for KRT in UAH, it is plausible that the FS’s true KRT access rate is close to Africa’s projected range. This dire situation highlights the need for RSA to invest in cost-effective ways to increase KRT access progressively; otherwise, efforts directed at early diagnosis would be futile in those who eventually progress to ESKD. Further, the early awareness of CKD and the realisation of limited access to KRT when needed produce an appreciable emotional toll on the health caregiver, patient, and family.

On logistic regression analysis, age and diabetes mellitus were shown to be independent predictors of exclusion from the KRT program. These two factors are associated with significant morbidity and mortality in patients on KRT, and thus carry an extra-economic burden on the health care system [42]. Thus, exclusion based on these factors avoids the added risk levied by them. This utilitarian outlook is one approach to addressing the ethical dilemma faced by many SSA countries of increasing health care demands in an environment with scarce prevailing resources. However, denying KRT access to those who may have contributed the most to society over the years while favouring the young who can still contribute economically is ethically challenging. Furthermore, as the FS and RSA KRT programme is based on transplantability with age no longer a widely recognised contraindication to transplantation globally, this exclusion factor is a contradiction [43]. Reports from transplant programmes with universal access show that older adults benefit significantly in terms of survival and quality of life from transplantation, with the average age of transplantation at above 60 years in many western countries [25, 44, 45]. Although some reports have noted increased mortality and hospitalisation rates associated with age, the reported graft and patient survival rates are more than 90 and 77 % at 1 and 5 years, respectively [46–48]. The age contradiction has persisted due to RSA’s resource constraints, as in many SSA countries.

Concomitant advanced and irreversible organ dysfunction, including cardiovascular and cardiopulmonary diseases, was the commonest reason for exclusion from the KRT programme. Cardiac disease and pulmonary hypertension were the commonest advanced organ dysfunctions cited as reasons for KRT access denial. Hypertension was identified as a significant predictor of concomitant advanced organ dysfunction resulting in exclusion. This association is likely a consequence of cardiovascular complications associated with hypertension. Likewise, cardiovascular complications in ESKD are associated with increased morbidity and mortality and can potentially impose additional socioeconomic risk on the health care system. Uncontrolled HIV, defined by unsuppressed viral load, was the second reason cited for denial of KRT. This exclusion is based on the high morbidity and mortality associated with uncontrolled HIV among ESKD patients on dialysis [49–51]. In 2016, the RSA government introduced a universal test-and-treat policy, making available antiretroviral treatment to all who test positive for HIV irrespective of CD4 count. This strategy is hoped to progressively mitigate against the complications, morbidity and mortality associated with HIV infection [52]. Likewise, these universal provisions should alleviate the presentation of patients with ESKD and uncontrolled HIV, as this strategy’s benefits are gradually realised.

Our study’s main limitation was that it was a single-centre retrospective study with several incomplete files being excluded in the analysis, thereby limiting the generalisability of the findings. Some of the incomplete files were of patients who died shortly after admission to the UAH before a decision on the eligibility for KRT could be made. The non-referral of some patients with ESKD to the UAH with obvious exclusion factors likely contributed to the underestimation of ESKD incidences and KRT access rates in the FS. However, this study’s strength is that the UAH is the only centre that processes KRT access for the entire FS province, allowing for KRT incidence rates of the province’s public sector to be readily attainable.

## Conclusions

The FS public health care sector serves a mostly young and economically able but indigent portion of the society. Both non-communicable and communicable diseases, including hypertension, diabetes, and HIV, contributed to the causes of ESKD. Although two-thirds of the patients are still excluded from accessing KRT, the FS provincial KRT incidence grew by 75 % between 2016 and 2018. Age, diabetes mellitus, and advanced organ dysfunction were significant factors for KRT access denial. Hypertension was the major independent risk factor for developing concomitant advanced organ dysfunction resulting in exclusion from dialysis access. All major ESKD aetiologies in our study were potentially preventable and manageable. Strategies aimed at early identification and prevention at the primary health care level may help alleviate some of the burdens imposed by ESKD on the RSA public health system. However, more resources are needed to meet the increasing demands for KRT. Likewise, more country-level studies are required to define further the epidemiology of CKD and contributors to ESKD in RSA.

## Supplementary Information

**Additional file 1: **

## Data Availability

The datasets used and/or analysed during the current study are available from the corresponding author on reasonable request.

## Abbreviations

ESKD: End-stage kidney disease
CI: Confidence interval
CKD: Chronic kidney disease
ERA-EDTA: European Renal Association – European Dialysis and Transplant Association
FS: Free State province
GFR: Glomerular filtration rate
HIV: Human immunodeficiency virus
IQR: Interquartile range
OR: Odds ratio
PD: Peritoneal dialysis
pmp: Per million population
KRT: Kidney replacement therapy
RSA: Republic of South Africa
SSA: Sub-Saharan Africa
UAH: Universitas Academic Hospital
USRDS: United States Renal Data System
WC: Western Cape province

## References

[1] Levey AS, Eckardt KU, Tsukamoto Y, Levin A, Coresh J, Rossert J (2005). Definition and classification of chronic kidney disease: a position statement from kidney disease: improving global outcomes (KDIGO). Kidney Int.

[2] Naicker S (2010). Burden of end-stage renal disease in sub-Saharan Africa. Clin Nephrol.

[3] Murphy D, McCulloch CE, Lin F, Banerjee T, Bragg-Gresham JL, Eberhardt MS (2016). Trends in prevalence of chronic kidney disease in the United States. Ann Intern Med..

[4] Zhang QL, Rothenbacher D (2008). Prevalence of chronic kidney disease in population-based studies: systematic review. BMC Public Health..

[5] Hill NR, Fatoba ST, Oke JL, Hirst JA, O’Callaghan CA, Lasserson DS (2016). Global prevalence of chronic kidney disease - a systematic review and meta-analysis. PLoS One.

[6] Hodel NC, Hamad A, Praehauser C, Mwangoka G, Kasella IM, Reither K (2018). The epidemiology of chronic kidney disease and the association with non-communicable and communicable disorders in a population of sub-Saharan Africa. PLoS One.

[7] Arogundade FA, Barsoum RS (2008). CKD prevention in Sub-Saharan Africa: a call for governmental, nongovernmental, and community support. Am J Kidney Dis.

[8] Stanifer JW, Jing B, Tolan S, Helmke N, Mukerjee R, Naicker S (2014). The epidemiology of chronic kidney disease in sub-Saharan Africa: a systematic review and meta-analysis. Lancet Glob Health.

[9] Mayosi BM, Flisher AJ, Lalloo UG, Sitas F, Tollman SM, Bradshaw D (2009). The burden of non-communicable diseases in South Africa. Lancet.

[10] Etheredge H, Fabian J (2017). Challenges in expanding access to dialysis in South Africa—expensive modalities, cost constraints and human rights. Healthcare.

[11] Kilonzo KG, Jones ESW, Okpechi IG, Wearne N, Barday Z, Swanepoel CR (2017). Disparities in dialysis allocation: An audit from the new South Africa. PLoS One.

[12] Wang V, Vilme H, Maciejewski ML, Boulware LE (2016). The economic burden of chronic kidney disease and end-stage renal disease. Semin Nephrol.

[13] Naicker S (2013). End-stage renal disease in Sub-Saharan Africa. Kidney Int Suppl.

[14] Liyanage T, Ninomiya T, Jha V, Neal B, Patrice HM, Okpechi I (2015). Worldwide access to treatment for end-stage kidney disease: a systematic review. Lancet.

[15] Hassen M, Archer E, Pellizzon A, Chikte UME, Davids MR (2020). Human resources for nephrology in South Africa: A mixed-methods study. PLoS One.

[16] Mayosi BM, Benatar SR (2014). Health and health care in South Africa–20 years after Mandela. N Engl J Med.

[17] Naicker S (2003). End-stage renal disease in sub-Saharan and South Africa. Kidney Int Suppl.

[18] Statistics SA: Census 2011 Census in brief. Statistics South Africa. 2012. http://www.statssa.gov.za/census/census_2011/census_products/Census_2011_Census_in_brief.pdf. Accessed 9 Feb 2021.

[19] Statistics SA: 2016–2018 Mid-year population estimates. Statistics South Africa. 2019. http://www.statssa.gov.za/. Accessed 5 Feb 2021.

[20] Statistics SA: 2016–2018 General Household Survey. Statistics South Africa. 2019. http://www.statssa.gov.za/. Accessed 5 Feb 2021.

[21] Moosa MR. Priority setting approach in the selection of patients in the public sector with end-stage kidney failure for renal replacement treatment in the Western Cape Province. 2013. http://s3.documentcloud.org/documents/19489/moosa-priority-setting-policy-final-feb-24-2010-final.pdf. Accessed on 2nd March 2021

[22] De Meyer V, Abramowicz D, De Meester J, Collart F, Bosmans JL, Cools W, et al. Variability in the incidence of kidney replacement therapy over time in Western industrialised countries: A retrospective registry analysis. PLoS One. 2020;15: e0235004. 10.1371/journal.pone.0235004 PMC7316284 32584849

[23] Gonzalez-Bedat M, Rosa-Diez G, Pecoits-Filho R, Ferreiro A, Garcia-Garcia G, Cusumano A (2015). Burden of disease: prevalence and incidence of ESRD in Latin America Clin Nephrol. Suppl.

[24] Alashek WA, McIntyre CW, Taal MW (2012). Epidemiology and aetiology of dialysis-treated end-stage kidney disease in Libya. BMC Nephrol.

[25] Kramer A, Boenink R, Noordzij M, Bosdriesz JR, Stel VS, Beltran P, et al. The ERA-EDTA Registry Annual Report 2017: a summary. Clin Kidney J. 2020;13:693–709. 10.1093/ckj/sfaa048 PMC7467580 32897277

[26] Moosa MR, Maree JD, Chirehwa MT, Benatar SR (2016). Use of the ‘accountability for reasonableness’ approach to improve fairness in accessing dialysis in a middle-income country. PLoS One..

[27] Statistics SA. Findings from the Living Conditions Surveys, 2008/2009–2014/2015. Statistics South Africa. 2017. http://www.statssa.gov.za/. Accessed 5 Feb 2021.

[28] Arora P, Obrador GT, Ruthazer R, Kausz AT, Meyer KB, Jenuleson CS (1999). Prevalence, predictors, and consequences of late nephrology referral at a tertiary care center. J Am Soc Nephrol.

[29] Navaneethan SD, Aloudat S, Singh S (2008). A systematic review of patient and health system characteristics associated with late referral in chronic kidney disease. BMC Nephrol.

[30] Lunney M, Alrukhaimi M, Ashuntantang GE, Bello AK, Bellorin-Font E, Benghanem Gharbi M (2018). Guidelines, policies, and barriers to kidney care: findings from a global survey. Kidney Int Suppl.

[31] Vanholder R, Annemans L, Brown E, Gansevoort R, Gout-Zwart JJ, Lameire N (2017). Reducing the costs of chronic kidney disease while delivering quality health care: a call to action. Nat Rev Nephrol.

[32] James MT, Hemmelgarn BR, Tonelli M (2010). Early recognition and prevention of chronic kidney disease. Lancet.

[33] Imai E, Yamagata K, Iseki K, Iso H, Horio M, Mkino H (2007). Kidney disease screening program in Japan: history, outcome, and perspectives. Clin J Am Soc Nephrol.

[34] Codreanu I, Perico N, Sharma SK, Schieppati A, Remuzzi G (2006). Prevention programmes of progressive renal disease in developing nations. Nephrology.

[35] Luyckx VA, Cherney DZI, Bello AK (2020). Preventing CKD in developed countries. Kidney Int Rep.

[36] Davids MR, Jardine T, Marais N, Jacobs JC, Sebastian S. South African renal registry annual report 2018. Afr J Nephrol. 2020;23:185 – 96.

[37] Saran R, Robinson B, Abbott KC, Bragg-Gresham J, Chen X, Gipson D, et al. US Renal Data System 2019 Annual data report: epidemiology of kidney disease in the United States. Am J Kidney Dis. 2020;Suppl 1:6–7. 10.1053/j.ajkd.2019.09.003 31704083

[38] International Diabetes Federation. IDF diabetes atlas 8th edition. 2017. https://www.diabetesatlas.org/upload/resources/previous/files/8/IDF_DA_8e-EN-final.pdf?fbclid=IwAR1dhyFGa3ZlDQngZlEOoDVenpcMPl49ox8Us8nWL9_rSv0-3PIWcZio8bY. Accessed on 2nd March 2021

[39] Mbanya JC, Motala AA, Sobngwi E, Assah FK, Enoru ST (2010). Diabetes in sub-Saharan Africa. Lancet.

[40] Mapa-Tassou C, Katte JC, Mba Maadjhou C, Mbanya JC (2019). Economic impact of diabetes in Africa. Curr Diab Rep.

[41] Anders HJ, Huber TB, Isermann B, Schiffer M (2018). CKD in diabetes: diabetic kidney disease versus nondiabetic kidney disease. Nat Rev Nephrol.

[42] Ma L, Zhao S (2017). Risk factors for mortality in patients undergoing hemodialysis: A systematic review and meta-analysis. Int J Cardiol.

[43] Chadban SJ, Ahn C, Axelrod DA, Foster BJ, Kasiske BL, Kher V, et al. KDIGO clinical practice guideline on the evaluation and management of candidates for kidney transplantation. Transplantation. 2020;Suppl 1:11–103. 10.1097/TP.0000000000003136 32301874

[44] Rao PS, Merion RM, Ashby VB, Port FK, Wolfe RA, Kayler LK (2007). Renal transplantation in elderly patients older than 70 years of age: results from the scientific registry of transplant recipients. Transplantation.

[45] Griva K, Davenport A, Harrison M, Newman SP (2012). The impact of treatment transitions between dialysis and transplantation on illness cognitions and quality of life–A prospective study. Br J Health Psychol.

[46] Dempster NJ, Ceresa CDL, Aitken E, Kingsmore D (2013). Outcomes following renal transplantation in older people: a retrospective cohort study. BMC Geriatr.

[47] Morales JM, Marcén R, del Castillo D, Andres A, Gonzalez-Molina M, Oppenheimer F (2012). Risk factors for graft loss and mortality after renal transplantation according to recipient age: a prospective multicentre study. Nephrol Dial Transplant.

[48] Knoll GA (2013). Kidney transplantation in the older adult. Am J Kidney Dis..

[49] Ndlovu KC, Sibanda W, Assounga A (2017). Peritonitis outcomes in patients with HIV and end-stage renal failure on peritoneal dialysis: a prospective cohort study. BMC Nephrol.

[50] Ndlovu KCZ, Assounga A (2017). Continuous Ambulatory Peritoneal Dialysis in Patients with HIV and End-Stage Renal Failure. Perit Dial Int.

[51] Rodriguez RA, Mendelson M, O’Hare AM, Hsu LC, Schoenfeld P (2003). Determinants of survival among HIV-infected chronic dialysis patients. J Am Soc Nephrol.

[52] Havlir D, Lockman S, Ayles H, Larmarange J, Chamie G, Gaolathe T (2020). What do the Universal Test and Treat trials tell us about the path to HIV epidemic control?. J Int AIDS Soc.

