# Supplementary material for: End-stage kidney disease and rationing of kidney replacement therapy in the free state province, South Africa: a retrospective study
Source: BMC Nephrol. 2021 May 11;22:174. doi: 10.1186/s12882-021-02387-x (PMC8112033; doi:10.1186/s12882-021-02387-x)
Supplement: Supplementary file 1 — Additional file 1: [file 12882_2021_2387_MOESM1_ESM.docx]

**Supplementary Table 1: Characteristics of patients with or without advanced organ dysfunction**

|  | Advanced organ dysfunction (n = 109) | No advanced organ dysfunction (n = 76) | *p*-value |
| --- | --- | --- | --- |
| Age (years), median (IQR) | 42 (33-49) | 44 (34.5-55) | 0.164 |
| Sex, n (%) | 44/109 (40.4) | 37/76 (48.7) | 0.262 |
| Hypertension, n (%) | 55/109 (50.5) | 22/76 (29.0) | 0.003 |
| Diabetes, n (%) | 26/109 (23.9) | 21/76 (27.6) | 0.561 |
| HIV, n (%) | 24/109 (22.0) | 31/76 (40.8) | 0.006 |
| SLE, n (%) | 4/109 (3.7) | 2/76 (2.6) | 1.000 |
| Smoking & alcohol, n (%) | 9/109 (8.3) | 5/76 (6.6) | 0.671 |
| Serum creatinine (μmol/L), median (IQR) | 778 (589-1100) | 896 (573-1237) | 0.398 |

HIV, human immunodeficiency virus; IQR, interquartile range; SLE, systemic lupus erythematosus
